# Supplementary material for: Quantitative Assessment of CD200 and CD200R Expression in Lung Cancer
Source: Cancers (Basel). 2021 Mar 1;13(5):1024. doi: 10.3390/cancers13051024 (PMC7957629; doi:10.3390/cancers13051024)
Supplement: Supplementary file 1 [file cancers-13-01024-s001.pdf]

cancers

MDPI

Supplemental Material

Quantitative Assessment of CD200 and CD200R Expression in Lung Cancer

Ioannis A. Vathiotis, Tyler MacNeil, Jon Zugazagoitia, Konstantinos N. Syrigos, Thazin Nwe Aung, Aaron M. Gruver, Peter Vaillancourt, Ina Hughes, Steve Hinton, Kyla Driscoll and David L. Rimm

Table S1. Overview of the three retrospective cohorts of lung cancer patients used in this study.

Cohort	Number of Tumors	Clinical Annotation	Histology	Tumor Genotype Available
Cohort 1 (YTMA423)	287	Yes	NSCLC	No
Cohort 2 (YMTA355)	30	No	LCNEC	No
Cohort 3 (YTMA310)	138	No	NSCLC	Yes
Total	455			

Table S2. Panel for multiplexed cytokeratin and CD200 immunofluorescence staining.

Target	Cytokeratin	CD200
Primary antibodies	Mouse polyclonal (Agilent), 1/100, 4 °C overnight incubation	Rabbit monoclonal IgG1 (clone 333, Sino Biological), 2.00 μg/mL, 4 °C overnight incubation
Secondary antibodies	Goat anti-mouse Alexa Fluor 546 (ThermoFisher Scientific), 1/100, RT 1 hour incubation	Anti-rabbit EnVision (K4009, Agilent), RT 1 hour incubation
Fluorescent reagents		Cy5-tyramide (Perkin-Elmer), RT 10 min incubation
Counterstain	DAPI, 1/1000, RT 5 min incubation	

Table S3. Panel for multiplexed cytokeratin and CD200R immunofluorescence staining.

Target	Cytokeratin	CD200R
Primary antibodies	Mouse polyclonal (Agilent), 1/100, 4 °C overnight incubation	Rabbit monoclonal IgG1 (Lilly Antibody 1, Eli Lilly and Company), 0.57 μg/ml, 4 °C overnight incubation
Secondary antibodies	Goat anti-mouse Alexa Fluor 546 (ThermoFisher Scientific), 1/100, RT 1 hour incubation	Anti-rabbit EnVision (K4009, Agilent), RT 1 hour incubation
Fluorescent reagents		Cy5-tyramide (Perkin-Elmer), RT 10 min incubation
Counterstain	DAPI, 1/1000, RT 5 min incubation	

Table S4. Panel for multiplexed cytokeratin and PD-L1 immunofluorescence staining.

Target	Cytokeratin	PD-L1
Primary antibodies	Mouse polyclonal (Agilent), 1/100, 4 °C overnight incubation	Rabbit monoclonal IgG (clone E1L3N, Cell Signaling Technology), 1 μg/mL, 4 °C overnight incubation
Secondary antibodies	Goat anti-mouse Alexa Fluor 546 (ThermoFisher Scientific), 1/100, RT 1 hour incubation	Anti-rabbit EnVision (K4009, Agilent), RT 1 hour incubation
Fluorescent reagents		Cy5-tyramide (Perkin-Elmer), RT 10 min incubation
Counterstain	DAPI, 1/1000, RT 5 min incubation	

Table S5. Anti-CD200 antibodies tested.

Vendor (Catalog Number)	Clonality	Clone Number	Host Species	Isotype	IF Assay Outcome
Abcam (ab23552)	Monoclonal	OX-104	Mouse	IgG1	Failed- Absence of membranous signal
Proteintech (66282-1-Ig)	Monoclonal	5F3D6	Mouse	IgG1	Fully Validated
Sino Biological (10886-R333)	Monoclonal	333	Rabbit	IgG	Fully Validated

Table S6. Anti-CD200R antibodies tested. Abbreviations; NA, not available.

Vendor (Catalog Number)	Clonality	Clone Number	Host Species	Isotype	IF Assay Outcome
Bio-Rad (MCA2282)	Monoclonal	OX108	Mouse	IgG1	Failed- Absence of membranous signal
Thermo Fisher Scientific (MA5-24119)	Monoclonal	380525	Mouse	IgG2B	Failed- Absence of membranous signal
Eli Lilly (NA)	Monoclonal	Lilly antibody 1	Rabbit	IgG1 kappa	Fully Validated
Eli Lilly (NA)	Monoclonal	Lilly antibody 2	Rabbit	IgG1 kappa	Fully Validated

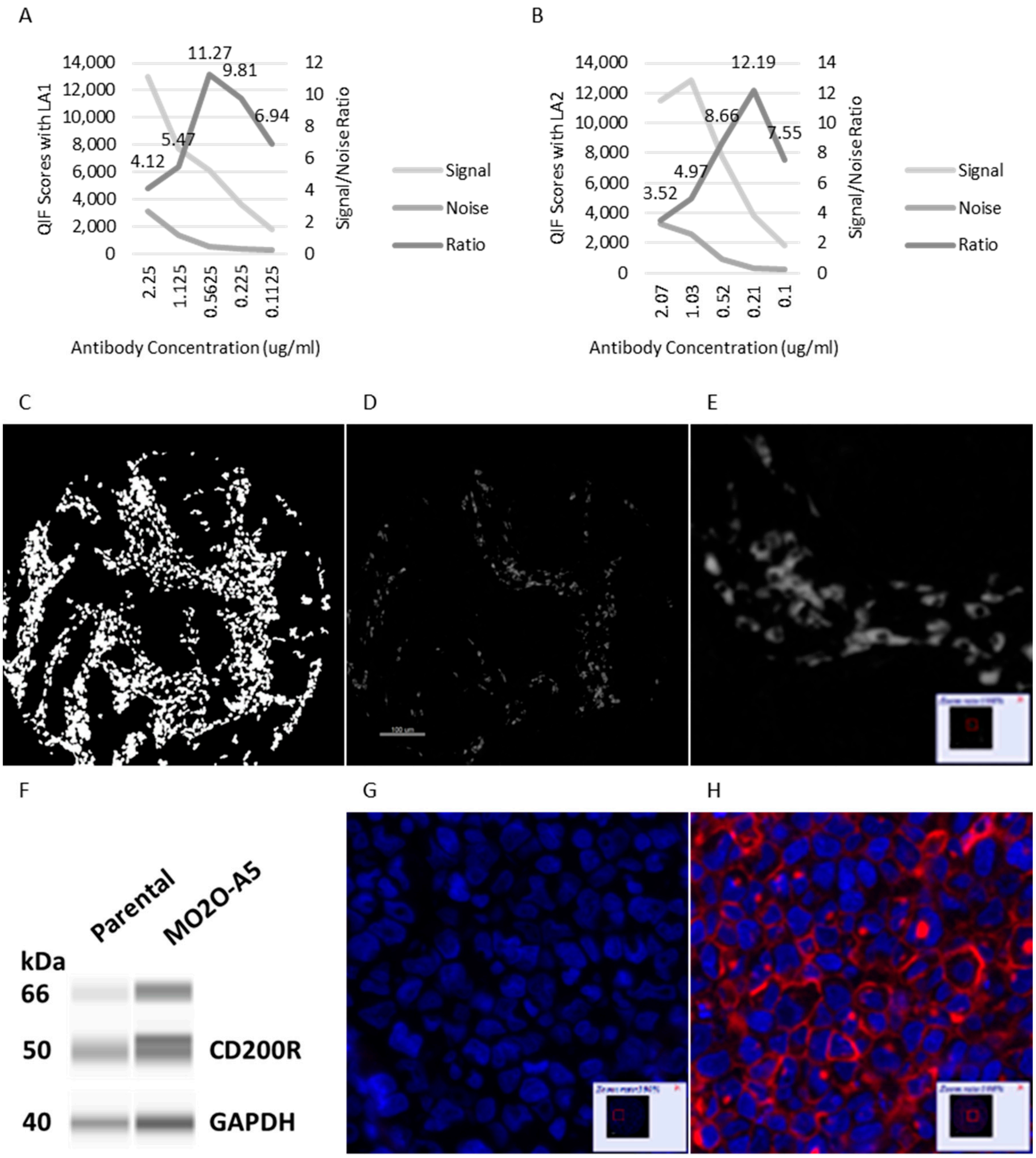
A
14,000
12,000
10,000
8,000
6,000
4,000
2,000
0
12
10
8
6
4
2
0
11.27
9.81
6.94
5.47
4.12
QIF Scores with LA1
Signal/Noise Ratio
2.25
1.125
0.5625
0.225
0.1125
Antibody Concentration (ug/ml)
Signal
Noise
Ratio
B
14,000
12,000
10,000
8,000
6,000
4,000
2,000
0
14
12
10
8
6
4
2
0
12.19
8.66
7.55
4.97
3.52
QIF Scores with LA2
Signal/Noise Ratio
2.07
1.03
0.52
0.21
0.1
Antibody Concentration (ug/ml)
Signal
Noise
Ratio
C
D
E
F
Parental
MO2O-A5
kDa
66
50
40
CD200R
GAPDH
G
H

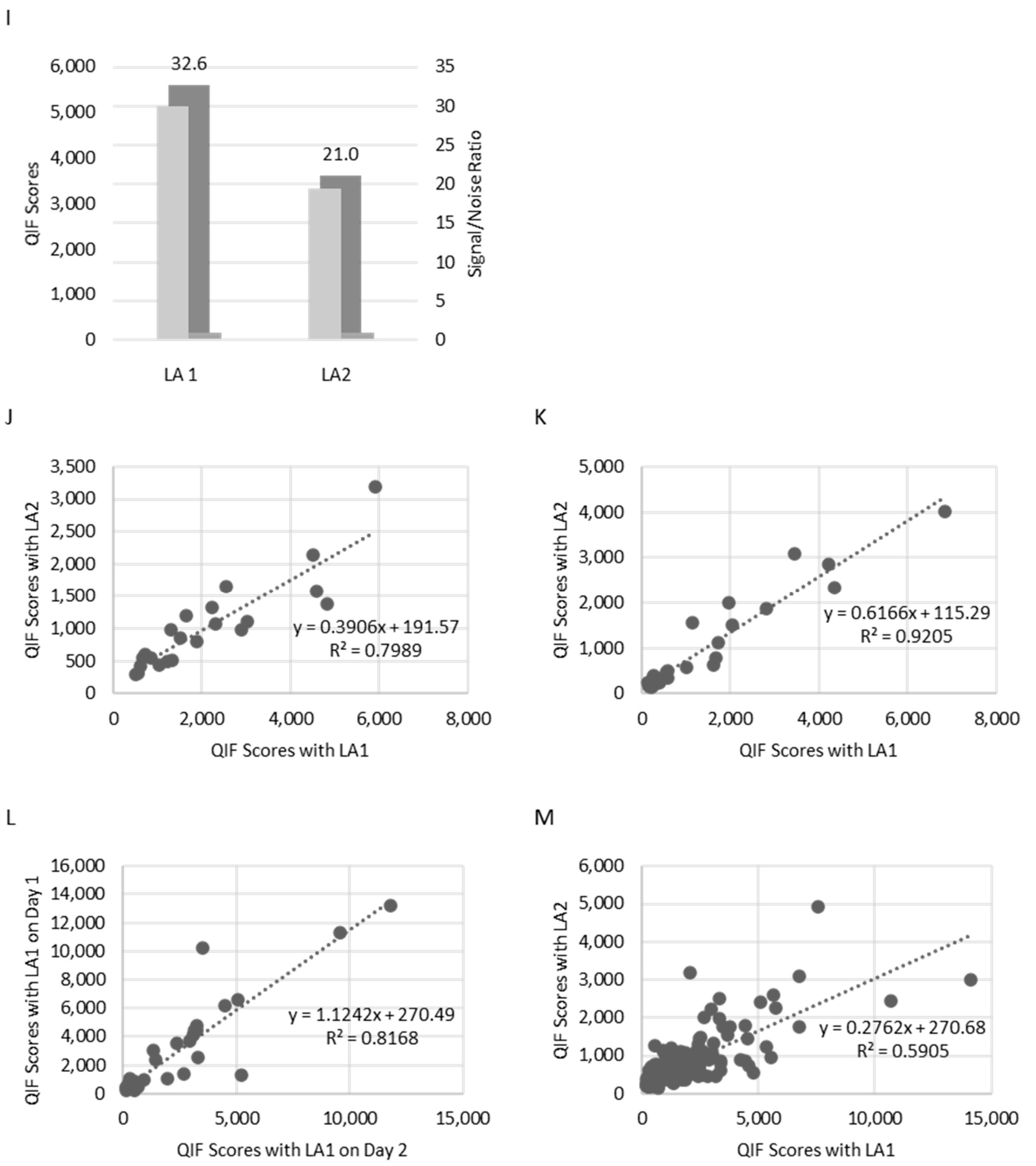

Figure S1. Validation of anti-CD200R Lilly Antibody 1. (**A**,**B**) Titration curves of two different anti-CD200R antibodies that were used for assay validation plotted at five different concentrations: Lilly Antibody 1 (LA1) (**A**) and Lilly Antibody 2 (LA2) (**B**); (**C**–**E**) Representative staining pattern for CD200R. Representative stromal mask of the spot (**C**). CD200R signal is localized within the stromal mask (**D**) with a membranous staining pattern (**E**). (**F**–**H**) Genetic methods of validation. Western blot showing CD200R protein expression levels for the CHO-parental and CHO-MO2O-A5 cell lines (**F**). Fluorescence staining for CD200R shows that signal is absent in the CHO-parental (**G**) but present in the CHO-MO2O-A5 cell line (H). Calculation of signal/noise ratio on YTMA424 favors LA1 over LA2 (**I**). (**J**–**M**) Cross-validation of anti-CD200R antibody clone LA1 with LA2 on YTMA295 (**J**) and YTMA424 (**K**). (**L**) Reproducibility of CD200R staining with clone LA1 in two different days on YTMA424. (**M**) Cross-validation of antibody LA1 with LA2 across different tumor types on YTMA395.

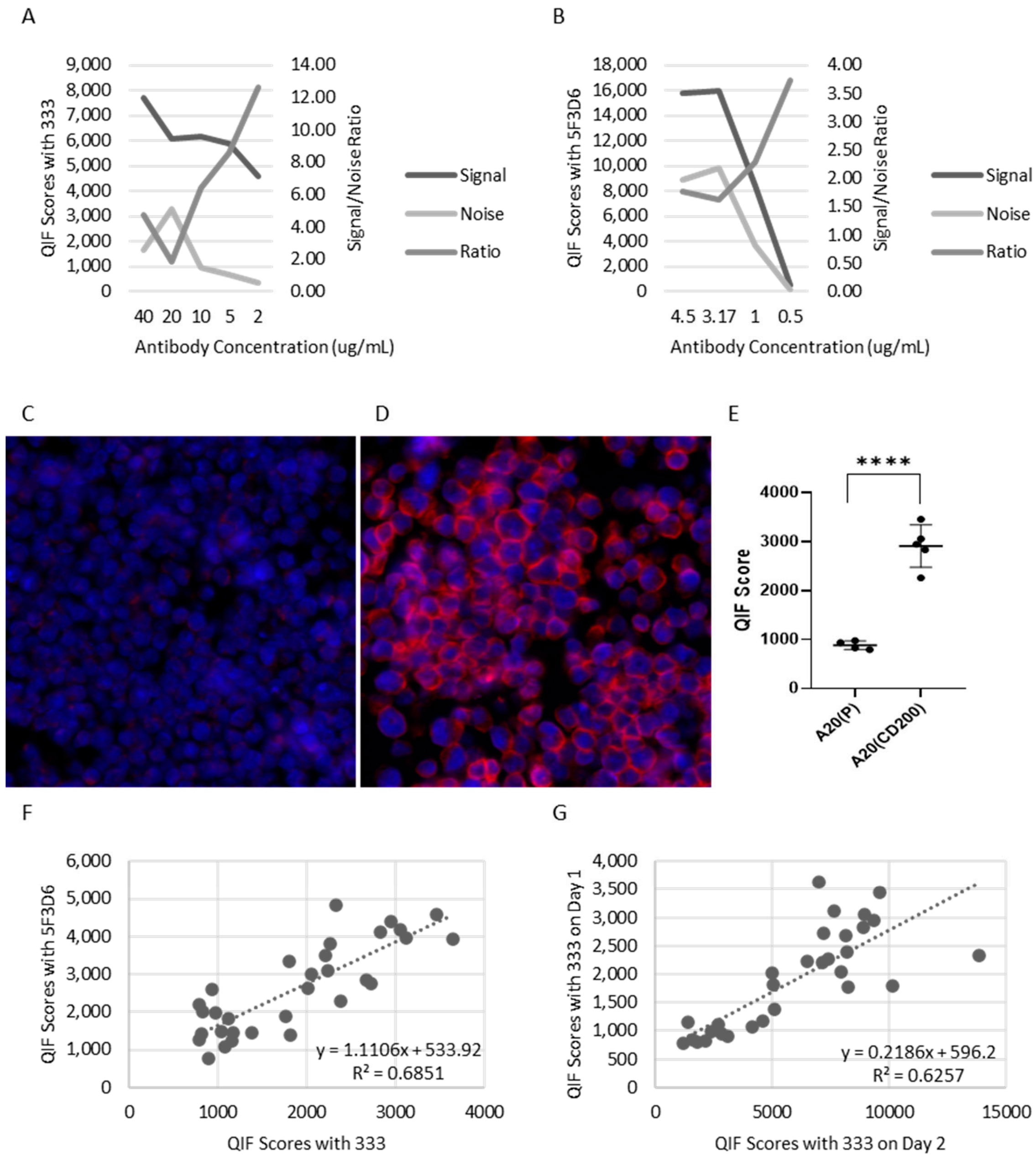

Figure S2. Validation of anti-CD200 antibody clone 333. (**A**,**B**) Titration curves of two different anti-CD200 antibodies that were used for assay validation plotted at five and four different concentrations, respectively: 333 (**A**) and 5F3D6 (**B**); (**C**–**E**) Genetic methods of validation. Fluorescence staining for CD200 shows that signal is low to absent in the A20 parental cell line (**C**) but high in the A20 CD200 overexpressing cell line (**D**). (**F**) Cross-validation of anti-CD200 antibody clone 333 with 5F3D6 on YTMA434. (**G**) Reproducibility of CD200 staining with clone 333 on two different days on YTMA434. ****, $p < 0.0001$.

A

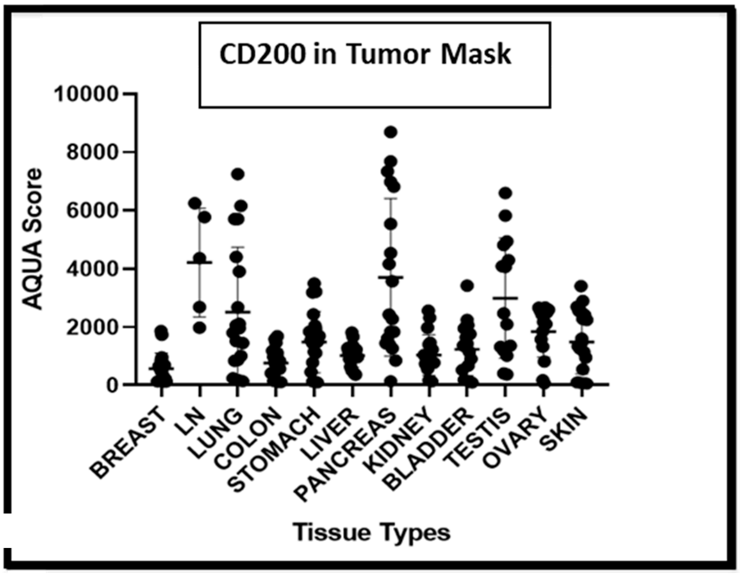

B

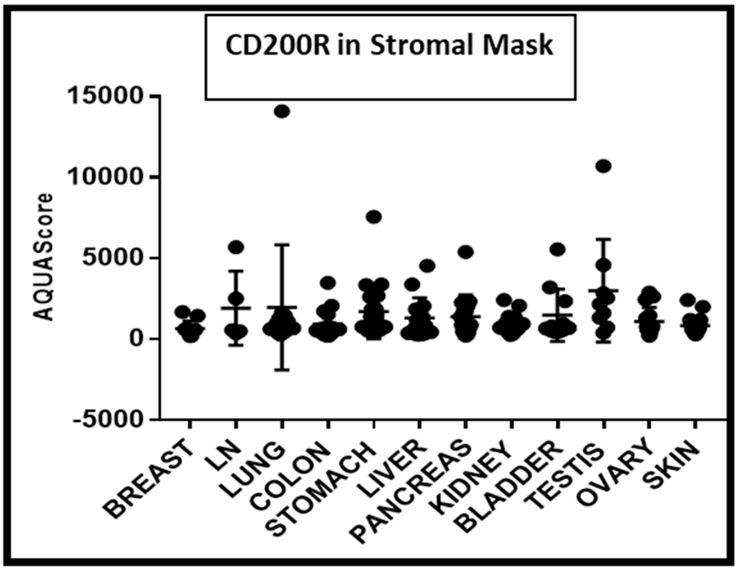

Figure S3. Expression of CD200 and CD200R across different tumor types. Quantitative immunofluorescence scores of CD200 measured in the tumor compartment (**A**) and CD200R measured in the stromal compartment (**B**) on YTMA395.

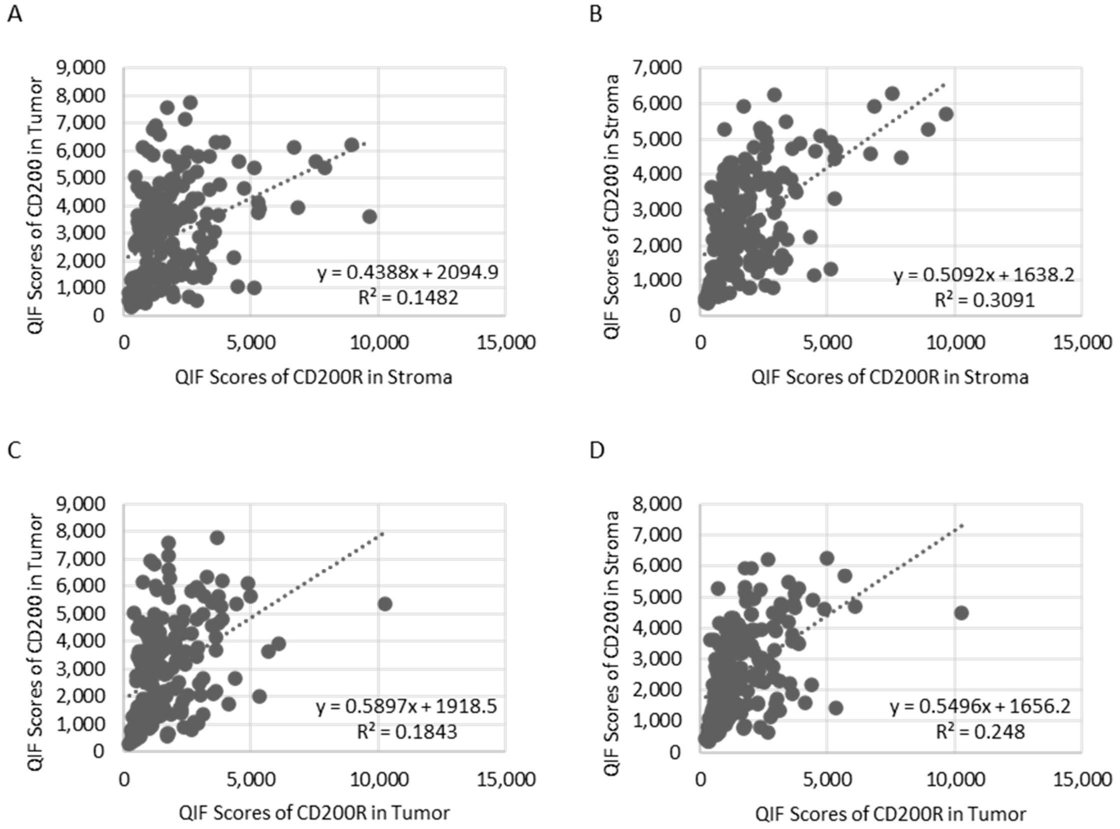

Figure S4. Correlation between CD200 and CD200R in NSCLC. (**A**,**B**) Scatter plots between CD200R measured in stroma and CD200 measured in tumor (**A**) and stroma (**B**). (**C**,**D**) Scatter plots between CD200R measured in tumor and CD200 measured in tumor (**C**) and stroma (**D**). Graphs were generated based on CD200 and CD200R expression on YTMA423.

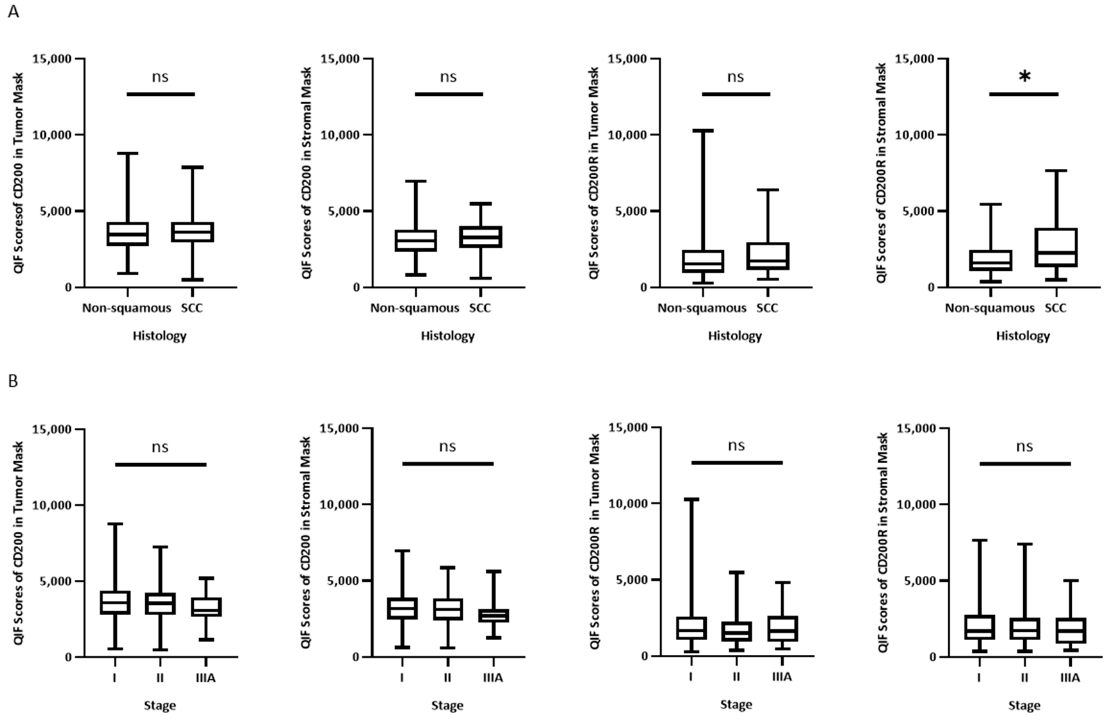

C

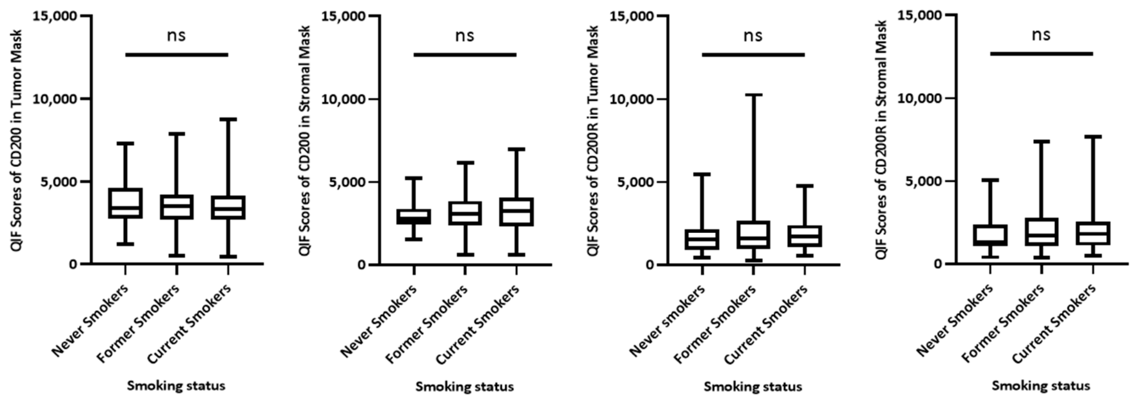

D

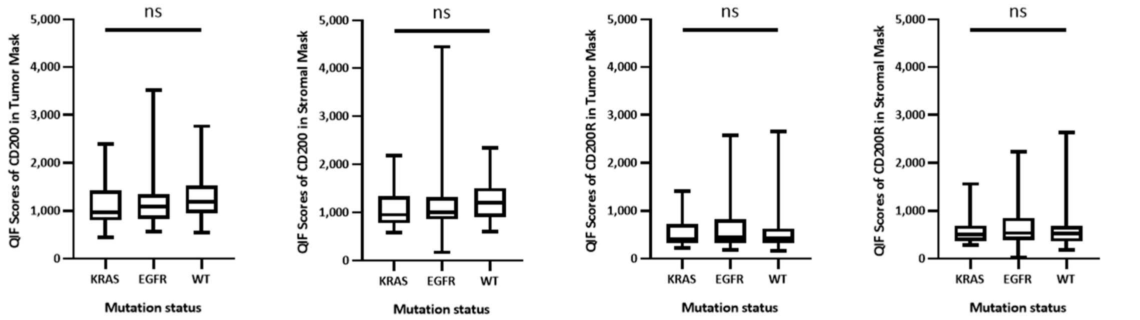

Figure S5. Association of CD200 and CD200R with clinicopathologic characteristics and genotype in NSCLC. (**A**–**C**) Association of CD200 and CD200R with histologic subtype (**A**), stage (**B**), smoking status (**C**) on YTMA 423. (**D**) Association of CD200 and CD200R with mutation status on YTMA310. Abbreviations; ns, not significant; *, $p < 0.05$.

A

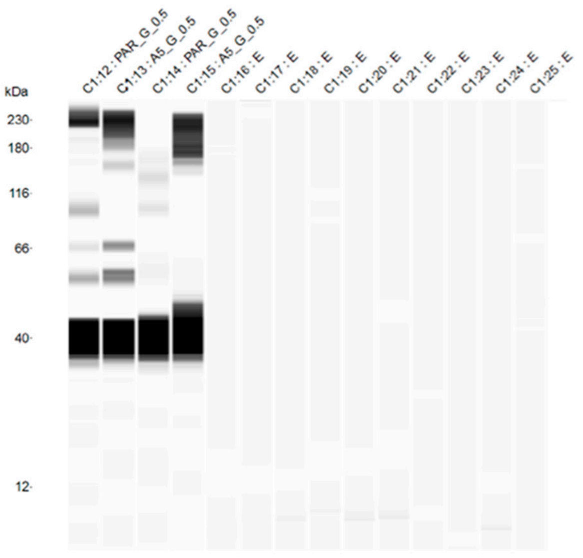

B

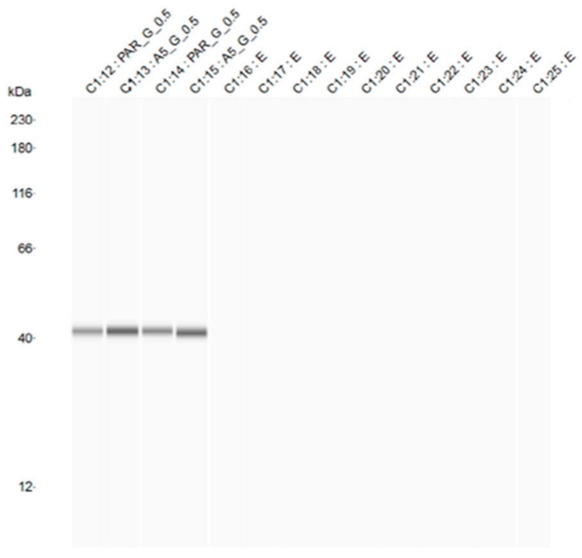

Figure S6. (**A**,**B**) Western blot in different exposure times for the identification of CD200R (**A**) and loading control GAPDH (**B**). Lane C1:12, CHO-parental cell line plus primary Lilly Antibody 1; lane C1:13, CHO-MO2O-A5 cell line plus primary Lilly Antibody 1; lane C1:14, CHO-parental cell line without primary antibody; lane C1:15, CHO-MO2O-A5 cell line without primary antibody; lanes C1:16 to C1:25 are empty.
